# Development of the Illinois Surgical Quality Improvement Collaborative (ISQIC): Implementing 21 Components to Catalyze Statewide Improvement in Surgical Care

**DOI:** 10.1097/AS9.0000000000000258

**Published:** 2023-03-01

**Authors:** Karl Y. Bilimoria, Michael F. McGee, Mark V. Williams, Julie K. Johnson, Amy L. Halverson, Kevin J. O’Leary, Paula Farrell, Juliana Thomas, Remi Love, Lindsey Kreutzer, Allison R. Dahlke, Brianna D’Orazio, Steven Reinhart, Katelyn Dienes, Mark Schumacher, Ying Shan, Christopher Quinn, Vivek N. Prachand, Susan Sullivan, Kimberly A. Cradock, Kelsi Boyd, William Hopkinson, Colleen Fairman, David Odell, Jonah J. Stulberg, Cindy Barnard, Jane Holl, Ryan P. Merkow, Anthony D. Yang

**Affiliations:** From the * Illinois Surgical Quality Improvement Collaborative (ISQIC) Coordinating Center, Chicago, IL; † Surgical Outcomes and Quality Improvement Center (SOQIC), Department of Surgery, Indiana University School of Medicine, Indianapolis, IN; ‡ Department of Internal Medicine at Washington University St. Louis, St. Louis, MO; § Division of Medicine-Hospital Medicine, Feinberg School of Medicine Northwestern University, Chicago, IL; ‖ Department of Process Improvement, Northwestern Medicine, Chicago, IL; ¶ Department of Surgery, University of Chicago Medicine, Chicago, IL; # Department of General Surgery, Carle Health, Urbana, IL; ** Department of Orthopaedic Surgery, Loyola University Medical Center, Maywood, IL; †† Department of Quality Strategies, Northwestern Medicine, Chicago, IL.

**Keywords:** benchmarking, education, guided implementation, quality improvement, Quality Improvement Collaborative, regional collaborative, surgery

## Abstract

**Introduction:**

In 2014, 56 Illinois hospitals came together to form a unique learning collaborative, the Illinois Surgical Quality Improvement Collaborative (ISQIC). Our objectives are to provide an overview of the first 3 years of ISQIC focused on (1) how the collaborative was formed and funded, (2) the 21 strategies implemented to support quality improvement (QI), (3) collaborative sustainment, and (4) how the collaborative acts as a platform for innovative QI research.

**Methods:**

ISQIC includes 21 components to facilitate QI that target the hospital, the surgical QI team, and the perioperative microsystem. The components were developed from available evidence, a detailed needs assessment of the hospitals, reviewing experiences from prior surgical and nonsurgical QI Collaboratives, and interviews with QI experts. The components comprise 5 domains: guided implementation (eg, mentors, coaches, statewide QI projects), education (eg, process improvement [PI] curriculum), hospital- and surgeon-level comparative performance reports (eg, process, outcomes, costs), networking (eg, forums to share QI experiences and best practices), and funding (eg, for the overall program, pilot grants, and bonus payments for improvement).

**Results:**

Through implementation of the 21 novel ISQIC components, hospitals were equipped to use their data to successfully implement QI initiatives and improve care. Formal (QI/PI) training, mentoring, and coaching were undertaken by the hospitals as they worked to implement solutions. Hospitals received funding for the program and were able to work together on statewide quality initiatives. Lessons learned at 1 hospital were shared with all participating hospitals through conferences, webinars, and toolkits to facilitate learning from each other with a common goal of making care better and safer for the surgical patient in Illinois. Over the first 3 years, surgical outcomes improved in Illinois.

**Discussion:**

The first 3 years of ISQIC improved care for surgical patients across Illinois and allowed hospitals to see the value of participating in a surgical QI learning collaborative without having to make the initial financial investment themselves. Given the strong support and buy-in from the hospitals, ISQIC has continued beyond the initial 3 years and continues to support QI across Illinois hospitals.

## BACKGROUND

Healthcare quality varies substantially among hospitals in the United States.^1–4^ In particular, surgical care results in considerable morbidity, mortality, and costs, with considerable variation in performance among U.S. hospitals.^5–8^ Several registries have been developed over the past 2 decades to improve quality by highlighting variation in quality and benchmarking hospitals with comparative performance reports, intending to identify targeted opportunities for improvement.^9,10^

In surgical care, the most prominent registry is the American College of Surgeons National Surgical Quality Improvement Program (ACS NSQIP), currently considered the gold standard for high-quality surgical data upon which to base quality improvement (QI) efforts.^11^ Launched by the ACS in 2004 after adapting the version available in the VA health system since the 1990s, ACS NSQIP has been implemented in more than 700 hospitals worldwide. ACS NSQIP is driven by the abstraction of clinical data specifically for the purpose of QI. Data are internally collected at each participating hospital by trained and audited data collectors using rigorously standardized data definitions.^10,12^ The data include each patient’s demographics, comorbidities, surgical details, and more than 30 postoperative outcomes, irrespective of whether the patient is still an inpatient, discharged, or readmitted. Hospitals are provided with benchmark comparison reports that allow each hospital opportunities to examine their performance with that of the other hospitals and identify opportunities for improvement. However, evaluations have demonstrated conflicting results regarding the effectiveness of ACS NSQIP in improving quality, particularly in a sustained fashion.^13–15^

While registries provide hospitals with an ability to measure quality, actually improving quality requires more complicated and nuanced efforts in planning, implementing, and monitoring resultant outcomes. To further facilitate improvement, groups of hospitals have also come together to form Quality Improvement Collaboratives (QICs) that work on common initiatives. QICs typically formed based on similar geography, specialization, or consonance of participant hospitals and have functioned to share, learn, and crowd-source QI experiences among participants while providing performance benchmarks among the peer group of hospitals.^16–21^ QICs have become particularly prevalent in surgical care.^22–25^ While some QICs have shown success in initially reducing complication rates and costs,^26–31^ there are growing concerns that simply meeting as a group and using a common measurement platform is not enough to achieve improved outcomes that are sustained over time.^13,14,32,33^

To address these concerns, the Illinois Surgical Quality Improvement Collaborative (ISQIC) was conceptualized in late 2014 to improve surgical quality through a novel holistic QI approach for a large group of Illinois hospitals. Following completion of a comprehensive needs assessment, 21 strategies were identified, and these strategies were implemented to help facilitate collaborative QI in ISQIC (Supplemental Table 1, http://links.lww.com/AOSO/A209). Our objectives are to provide an overview of the first 3 years of ISQIC focused on (1) how the collaborative was formed and funded, (2) the 21 strategies implemented to support QI, (3) statewide initiatives undertaken to date, (4) collaborative sustainment, and (5) how the collaborative acts as a platform for innovative QI research.

## DEVELOPMENT OF ISQIC

### Conceptualization and Needs Assessment

A group of Illinois surgeons and QI professionals interested in surgical QI informally convened in 2012 to discuss development of a regional surgical QIC for Illinois. Moreover, we met with numerous quality collaborative leaders and QI experts from around the country to determine how ISQIC could be designed to address common issues that typically stymie QI efforts, particularly in surgical care and particularly in prior ACS NSQIP QICs. Following that, in 2013, a detailed needs assessment was conducted to characterize barriers and gaps that have historically hindered QI efforts at Illinois hospitals. The assessment was conducted through a series of interviews and surveys with the goal of (1) characterizing QI resources available at each hospital, (2) determining barriers to QI at each hospital, (3) assessing lessons learned from prior surgical and nonsurgical QICs, (4) conducting interviews/focus groups/surveys with surgeons, nurses, and administrators at Illinois hospitals, and (5) undertaking interviews with QI experts, leaders of other QICs, and local ACS chapter leaders.^26,28,29,34–36^

The needs assessment identified several key opportunities that could be leveraged into ISQIC’s early unique design. Primarily, hospitals expressed concerns that they were unsure if the startup costs of joining ACS NSQIP (>$110,000 year minimum for annual fee and data abstractor) were worth it. Second, surgeons reported they received very little formal training in QI and process improvement (PI) approaches. Resultantly, surgeons had little guidance about how to enact change when they found poor performance in a particular area. Third, surgeons were often unsure how to run the ACS NSQIP program, how to lead QI, or how to project manage these initiatives, and they believed guidance from a peer mentor could be essential for a collaborative’s success. Fourth, most hospitals had sparse personnel and funding to meaningfully improve quality in response to their comparative quality performance data when an issue was identified. Examining the issue, diving into the data, and developing interventions was time consuming and a real challenge for many hospitals. Finally, interviewees were hopeful that initial QIs can be made, but lacked experience in producing sustainable change.

Following the needs assessment of potential Illinois hospital participants, ISQIC leaders sought to gain experiential advice from existing QICs and national quality leaders. At the time of ISQIC’s conceptualization, approximately 20 collaboratives existed using the ACS NSQIP as the data platform. Interviews and conversations with selected QIC leaders and hospitals participating in these collaboratives reported the collaborative benefitted most from the shared learning opportunities and development of best practices. They have also been found to have greater improvement in outcomes and achieve these improvements faster than hospitals participating in ACS NSQIP alone. Additionally, collaborating hospitals demonstrated tremendous cost savings that outweighed ACS NSQIP participation costs.^10^ While existing collaboratives had demonstrated success by implementing ACS NSQIP and working collaboratively, ISQIC leaders identified several other opportunities to further improve the implementation, utility, and sustainability of a statewide surgical QI initiative.^26,28,29,34–36^

### Mission

ISQIC leaders sought to design ISQIC to leverage the strengths and experience of existing collaboratives to build a unique collaborative that targeted key gaps and needs for its constituent hospitals. ISQIC’s ultimate mission was chartered to improve the quality of surgical care in Illinois by providing hospitals the tools to (1) identify opportunities for QI using high-quality data, (2) examine areas of poor performance using a formal PI methodology, and (3) design and enact solutions to achieve the common goal of making care better and safer for surgical patients in Illinois. The focus of the collaboration is on reducing complication rates, mortality, length of stay, reoperations, and readmissions, while increasing adherence to best practice measures in strategically targeted areas. Moreover, there was an a priori focus on improving the culture of surgical quality at participating hospitals.

### Funding

After developing ISQIC’s mission, achieving funding to operationalize ISQIC’s mission was the paramount initial focus for ISQIC founders. The founders posited that surgical QIs for ISQIC hospitals would result in meaningful savings for hospitals and payers alike. While the estimated cost savings from QI vary considerably, previous studies have shown that the payer reaps approximately half of the cost savings attributed to QI efforts, while hospitals garner the other half.^5,6^ In 2010, the Patient Protection and Affordable Care Act was enacted and included a Medical Loss Ratio provision which required most health insurance to spend at least 80% of premium income on health care claims and QI, given the predicted influx of patients from the exchanges and resulting profits with the Affordable Care Act. The Medical Loss Ratio ensured that insurers could only achieve certain levels of profit from certain types of products.^37^ The provision effectively required that excess payor profit be returned to patients or purchasers, or they could be used to support hospitals in QI efforts as a quality bonus. ISQIC proposed a plan that utilized the insurer’s medical loss ratio mandate to develop funding for ISQIC. Blue Cross Blue Shield of Illinois (BCBS-IL) was the dominant payor in IL, with ~70% penetrance statewide. BCBS-IL would fund the first 3 years of ISQIC, at which point funding would be reevaluated. However, participant hospitals had to have an active fee-for-service contract with BCBS-IL to be eligible for ISQIC funding. Hospitals already engaged in predominantly managed care contracts with BCBS-IL were ineligible as the payer considered these hospitals to be already incentivized for providing high quality care through existing payment incentives (ie, quality activities were included in these contracts and hospitals were paid a fixed amount, so money saved would predominantly benefit the hospitals). Hospitals would be required to meet a number of participation and performance benchmarks to earn the funding each year. Of note, this type of funding may not be available in many states that do not have dominant payer; however, we believe that most of the initiatives we installed could be replicated elsewhere except for the funding of the hospital costs (eg, data abstractor, annual fee).

### Hospital Recruitment

Following conceptualization and funding securement, ISQIC was formally launched in 2014 as a collaborative effort between the Northwestern University Surgical Outcomes and Quality Improvement Center as the coordinating center, ACS NSQIP, the American College of Surgeons Metro Chicago and Illinois Chapters, and BCBS-IL. All Illinois hospitals that performed more than 5000 surgeries annually and had a contract for reimbursement with BCBS-IL were eligible for recruitment. The ISQIC coordinating center sent solicitation letters to existing Illinois ACS NSQIP Surgeon Champions (SCs) (ie, 9 Illinois hospitals were already enrolled in the registry), Surgical Clinical Reviewers (SCRs), and hospital administrators, as well as to all surgeons across Illinois. Prospective hospitals were directed to a website (www.ISQIC.org),^38^ to learn more about ISQIC and facilitate the application process. Applicants were required to complete a general information page about their hospital’s surgical volume, an assessment of current QI resources and programs, and 2 letters of executive support from hospital administration. All Illinois hospitals were solicited to participate in ISQIC, whether or not they participated in the ACS NSQIP at the time of recruitment. Certain hospitals that did not have an eligible fee-for-service contract with BCBS-IL were also allowed to participate in ISQIC without additional funding provided to the hospital specific to ISQIC. In addition, the 3 children’s hospitals in Illinois could apply to join ISQIC. Although ISQIC had initially budgeted and planned for approximately 25 hospital participants, interested exceeded expectations and BCBS-IL broadened the project scope and budget to allow any interested IL to participate.

**FIGURE 1.**
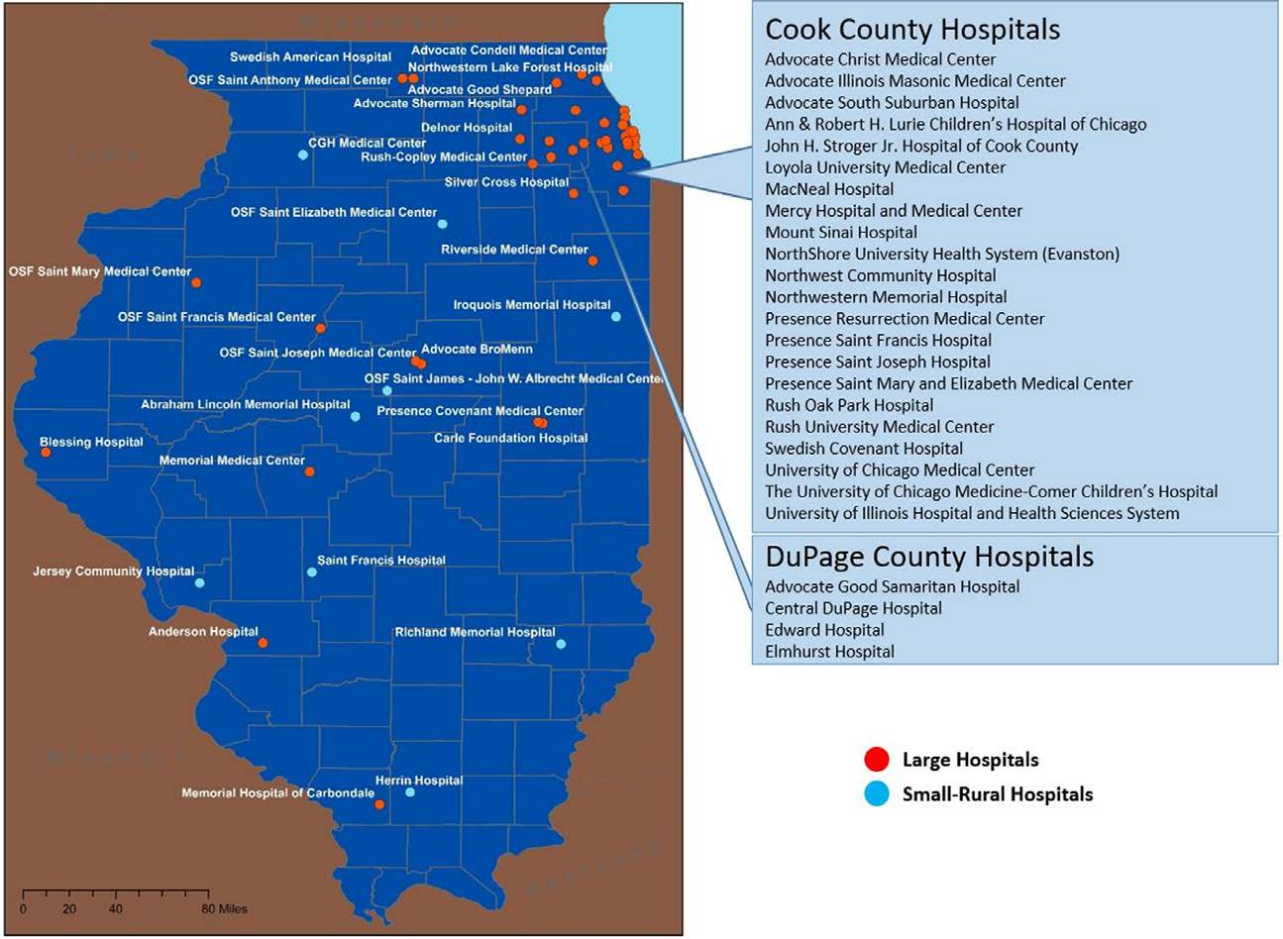
ISQIC hospital map.

During the initial recruitment period of 2013–2014, 46 hospitals contracted to participate in ISQIC (Fig. 1). ISQIC enrolled 27 hospitals that were not previously participating in ACS NSQIP prior to ISQIC and 9 that were participating in ACS NSQIP prior to ISQIC’s formation. These 36 hospitals were eligible to receive funding support to participate in ISQIC. The remaining 10 hospitals that were contractually ineligible to receive ISQIC/BSBL-IL funding, due to different contracts with BCBS-IL, also still joined ISQIC. Following this initial enrollment, ISQIC included every major academic medical center in Illinois and a diverse group of community hospitals.

Due to the overwhelming levels of interest from the initial ISQIC recruitment, ISQIC conducted a second enrollment targeting an expanded pool of small and rural Illinois hospitals that failed to meet requisite size criteria of the first solicitation. Ten additional small and/or rural hospitals enrolled in ISQIC (Fig. 1) and received funding from BCBS-IL, although minor customizations in participation and funding specific to the needs of these hospitals were created (ie, funded at a lower level with fewer requirements tied to earning the funding). Since small/rural hospitals perform largely outpatient and low complexity procedures, quality initiatives would focus on different topics, and financial stipends were reduced.

### ISQIC Coordinating Center and Launch

The ISQIC coordinating center was created to handle core operations of the geographically dispersed collaborative. The coordinating center included the ISQIC director, operations manager, program managers, research manager, QI and PI coaches, and other adjunct surgeons who lead specific initiatives for ISQIC. The coordinating center oversaw all efforts related to the operations, administration, research, and evaluation of ISQIC. In addition to serving as the point of contact for all local ISQIC hospital teams, the coordinating center handled external affairs regarding correspondence and negotiation with ACS, BCBS-IL, scholarly and lay media, and managed regulatory compliance and data integrity.

The ISQIC Advisory Committee was formed in parallel with hospital recruitment. The Committee consisted of members from participating ISQIC hospitals (large and small) including surgeons, nurses, and hospital administrators; ISQIC coordinating center staff; and a BCBS-IL representative. The advisory committee members represented all the different hospital types and locations in Illinois. The committee assisted with recruitment of hospitals and guided all aspects of ISQIC governance throughout the years of the collaborative by establishing goals, selecting statewide projects, participating in research, and awarding pilot grants. The advisory committee was chartered to meet at least twice yearly and on an ad hoc basis.

ISQIC was launched on September 1, 2014, with 56 hospitals (Fig. 1). At conception, ISQIC hospitals impacted more than 630,000 Illinois patients annually, performed 60% of all surgeries in Illinois, and performed over 80% of all inpatient surgeries in Illinois (Table 1). The Northwestern University Institutional Review Board office deemed ISQIC to be exempt.

**TABLE 1.** ISQIC Hospital Characteristics

ISQIC Hospital Characteristics			
	Large		Small/Rural
	**47 (83.9**)		**9 (16.1**)
NSQIP*		NSQIP*	
Experienced	19 (40.4)	Experienced	
New	28 (59.6)	New	9 (100)
Affiliated with hospital system		Affiliated with hospital system	
Yes	75%	Yes	56%
No	25%	No	44%
Teaching hospital			
Yes	68%		
No	32%		
Surgical cases		Surgical cases	
1000–4999	2%	0–1999	44%
5000–9999	39%	2000–2999	22%
10,000–14,999	32%	3000+	33%
15,000–19,999	11%	Average per year	2473
20,000–24,999	7%		
25,000+	9%		
Average per year	13,163		
Bed size		Bed size	
<100	4%	0–49	44%
100–299	50%	50–99	44%
300–499	30%	100+	12%
≥500	16%	Average bed size	62
Average bed size	329		

*Experienced: in ACS NSQIP prior to ISQIC initiation; new: joined ACS NSQIP and ISQIC in 2014.

## NOVEL APPROACHES FOR FACILITATING QUALITY IMPROVEMENT

Based on the needs assessment and evaluation of prior QICs, ISQIC founders developed an organizational structure for local hospital QI teams and a conceptual model for the collaborative to catalyze QI through 21 strategies organized into 5 domains,^15^ (Supplemental Table 1, http://links.lww.com/AOSO/A209): (1) guided implementation, (2) education and training, (3) comparative reports, (4) networking, and (5) financial support. The plan embraced a foundational understanding that each hospital has varying local needs specific to hospital characteristics (eg, size, resources, experience, patient population) and other less tangible contextual, environmental, and cultural factors. The ISQIC conceptual model afforded each hospital QI team flexibility to tailor their local QI plan to suit their specific needs.

### Hospital QI Teams

Each hospital assembled a local QI team including a SC, SCR, and a QI leader or administrator to implement and guide collaborative QI efforts at their respective hospital (Supplemental Figure 1, http://links.lww.com/AOSO/A209).

#### Surgeon Champion

The ISQIC SC is also the ACS NSQIP SC for the hospital. The SC is nominated locally by hospital leaders to lead surgical QI efforts for the hospital. ISQIC provided SCs a small annual stipend to support their effort, contingent upon meeting participation requirements. In addition to overseeing local ISQIC initiatives, the SC was required to convene monthly meetings with the SCR(s). These meetings were intended to allow local teams to communicate regularly about case abstraction, project updates, QI implementation, teamwork, and culture.

#### Surgical Clinical Reviewer

The SCR is the heart of the hospital QI team by functioning as both a data abstractor and project manager. Traditionally in ACS NSQIP, SCRs were just abstractors, but ISQIC focused on building project management into their portfolio as they were invested in the program and hospitals often struggled to find project managers for improvement initiatives. Prior to joining ISQIC, SCRs completed an intensive training program through ACS NSQIP and undergo annual continuing education didactics and data integrity audits. SCRs abstract clinical data that are submitted to ISQIC and ACS NSQIP that form the basis of ISQIC projects and benchmarking reports. SCRs functioned as the primary point of contact for the ISQIC coordinating center and ensure compliance with ISQIC deliverables and congruence with ISQIC objectives.

#### Quality Improvement Leader

The QI leader was a hospital administrative or operational leader with knowledge of the hospital’s QI efforts and access to local QI resources. The QI leader’s previous training and experience varied from hospital to hospital and ranged from hospital business administrators to formally trained project managers and performance improvement professionals. The QI leader facilitated the integration of the hospital’s quality team into the broader work of the collaborative.

#### Patient Safety Organization

ISQIC formally became an Agency for Healthcare Research and Quality federal Patient Safety Organization (PSO). The intent was to protect the data reports and “patient safety work product.” Of note, only the reports were protected as the PSO does not offer any protection for things already found in the medical record. In recent litigation, it was thought that the PSO did provide protections beyond state laws. However, the incredible administrative burden placed on hospitals and the ISQIC Coordinating Center made the PSO untenable. The requirements were arcane, and only 6 hospitals of 56 joined the PSO, thus rendering it useless. Even decommissioning the PSO ended up being an incredibly complicated process. Thus, we would not recommend that future collaboratives take the PSO route.

### Domain 1: Education and Training

#### Formal QI/PI Curriculum

**FIGURE 2.**
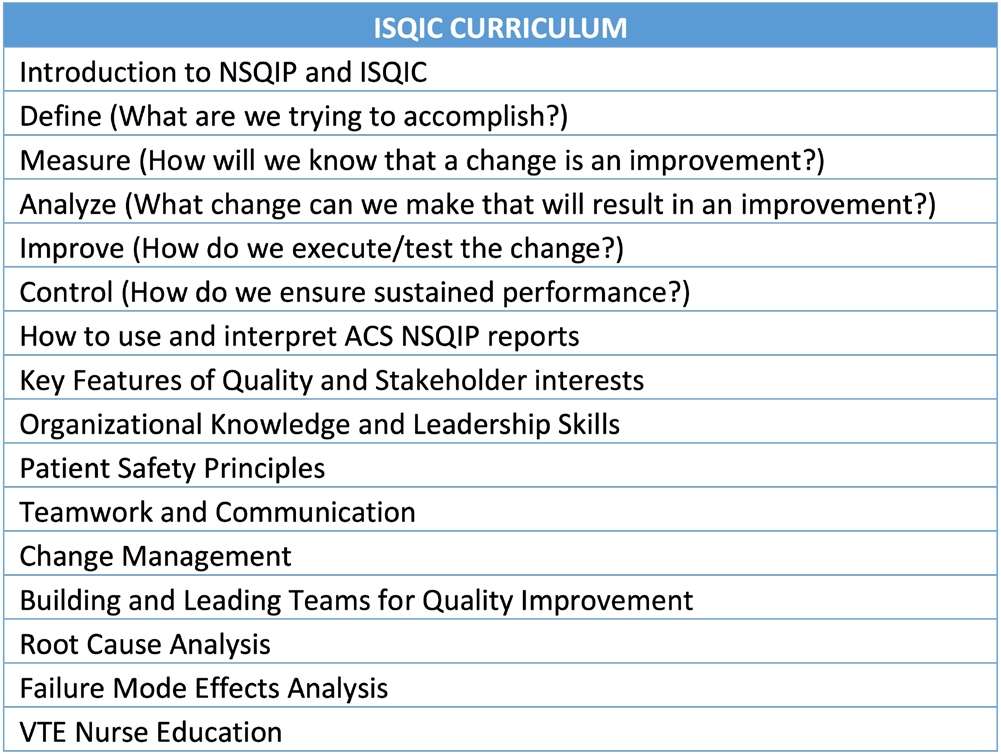
ISQIC training modules.

Early in the formation of ISQIC, a formal educational and training curriculum was developed for participants to strengthen PI knowledge. The content came from a combination of materials from established organizations, from our own PI academy, and developed de novo by process and QI experts on our team and in partnership with international experts. ISQIC hospital teams were required to complete training on (1) understanding quality and stakeholder interests, (2) organizational knowledge and leadership skills, (3) patient safety principles, (4) teamwork and communication, and (5) leadership and change management. The training consisted of twelve 20–40-minute, online modules that were bolstered with in-person didactic training twice yearly held during ISQIC semiannual conferences to consolidate the learning (Fig. 2). The modules topics included: Define, Measure, Analyze, Improve, Control (DMAIC) curriculum (1 module for each step), how to use clinical data for QI, building and leading teams for QI, root cause analysis, failure mode effects analysis, and project management.

**FIGURE 3.**
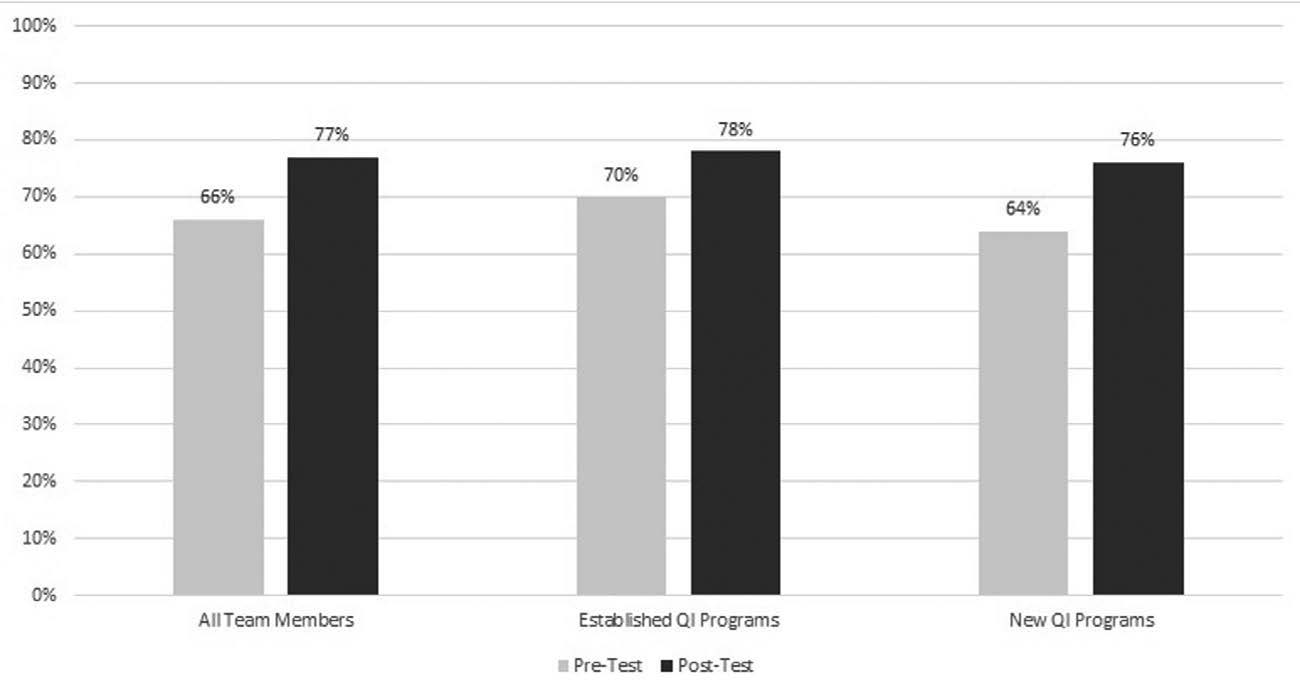
Improvement in QI-KAT with ISQIC.

The ISQIC coordinating center adapted the Quality Improvement Knowledge Application Tool (QIKAT) to assess the impact of surgical QI training and knowledge of performance improvement principles.^39^ Pre-curriculum QIKAT assessments allowed the coordinating center to intensify specific curriculum aspects to meet individual hospital needs. Following curriculum completion, 162 participants from 52 ISQIC hospital statistically improved their QIKAT scores (pre-test 66%, post-test 77%; *P* < 0.05) (Fig. 3). While the ISQIC curriculum was effective for all local team roles, the curriculum provided the most benefit for SCRs. Hospitals who were new to QI demonstrated the best knowledge growth following education. Importantly, the curriculum moved the group more than halfway toward mastery of QI and PI concepts in an expedited fashion. To the authors’ knowledge, this is the first statewide surgical QIC to implement and assess a formal QI education for members of the hospital team.

#### Data Management Training

As part of initial training, an intermediate level in-person Microsoft Excel class was offered by the ISQIC PI coaches to familiarize hospital teams with manipulating their data to create simple reports to aid QI projects. Excel training was provided in response to hospital teams’ initial concerns regarding data management and basic statistical analysis. Participants used actual ISQIC process measure data to learn arithmetic functions, sort and filter data, and create dynamic (eg, “pivot”) data tables for sorting and analysis. Additional exercises were made available after the class to reinforce skills and provide more examples of how to provide high quality data and reports back to their hospital groups. In-person advanced Excel training continued at semiannual conferences. The course was repeated, each time adding more complexity through the next 3 years.

#### Hospital Board and Leadership Engagement

The initial ISQIC needs assessment highlighted potential difficulties of garnering local hospital resources for QI. To this end, an ISQIC requirement was that the hospital’s ISQIC performance had to be presented to the hospital’s Board of Directors. ISQIC developed and provided hospital QI teams with turnkey materials intended to engage executive support with ISQIC initiatives. PowerPoint, email, letter templates, and “elevator pitch” speaking points were provided for SC s to use locally to engage hospital leadership and motivate support for ISQIC initiatives.

### Domain 2: Comparative Reports

#### Common Data Platform

All ISQIC hospitals utilized ACS NSQIP as the core requisite data collection platform. ACS NSQIP data is comprised of patient-level perioperative factors, yet notably absent from the platform is accounting of care delivery process measures that ISQIC QI projects required. To bridge the data gap between ACS NSQIP and ISQIC care delivery projects, the coordinating center created a complimentary proprietary secure data platform which enabled flexible collection of detailed process measure data. The ISQIC data platform (www.ISQICdata.org),^40^ incorporated logic algorithms into process measure abstraction, whereby improving SCRs abstraction efficiency (Supplemental Figure 2, http://links.lww.com/AOSO/A209). To ensure that mandated ACS NSQIP case abstraction minimums do not detract from the time necessary to complete additional ISQIC initiatives, ISQIC successfully petitioned ACS NSQIP to decrease the minimum number of required cases for ISQIC hospitals from 1680 to 1300 per year. Of note, as the collaborative became more mature, many hospitals felt they could more readily engage in QI with just the ISQIC Data Platform and the focus on process measures. Thus, after the 3-year introductory period, they often dropped out of ACS NSQIP and participated in ISQIC through the ISQIC Data Platform alone.

#### Comparative Performance Reports

**FIGURE 4.**
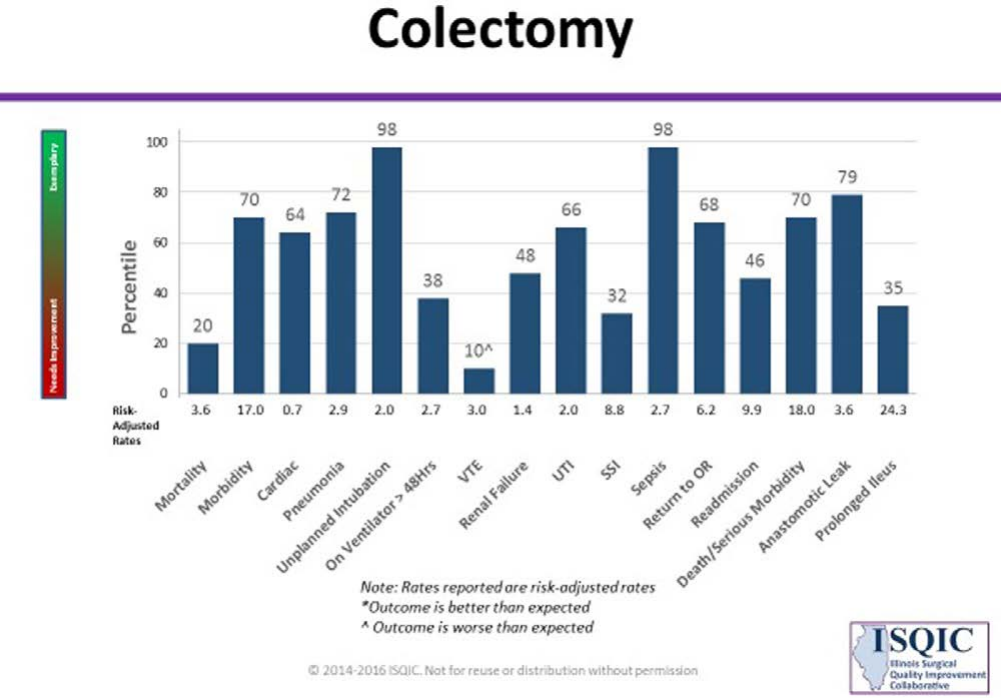
ISQIC benchmarked hospital quality reports. UTI indicates urinary tract infection. OR, Operating Room.

Early in ISQIC’s development, hospitals noted that standard ACS NSQIP performance reports were challenging to understand pragmatically at a local QI level which hindered direct use in identifying and guiding local QI efforts. Thus, ISQIC created specialized semiannual reports using a new format developed through an iterative process and feedback from Illinois hospitals and surgeons (Fig. 4). In addition to the national outcome reports that each hospital receives from ACS NSQIP, ISQIC hospitals also received reports containing risk-adjusted benchmarked surgical outcomes of each hospital’s performance relative to other ISQIC hospitals. ISQIC benchmarked reports also included granular data regarding process measure adherence for ISQIC initiatives (eg, surgical site infection [SSI] and venous thromboembolism [VTE] Collaborative Quality Improvement Projects [CQIPs]). ISQIC benchmarking reports were developed and iteratively revised over the course of the first 3 years based on ISQIC hospital feedback.

#### Comparative Data on Surgical Safety Culture

**FIGURE 5.**
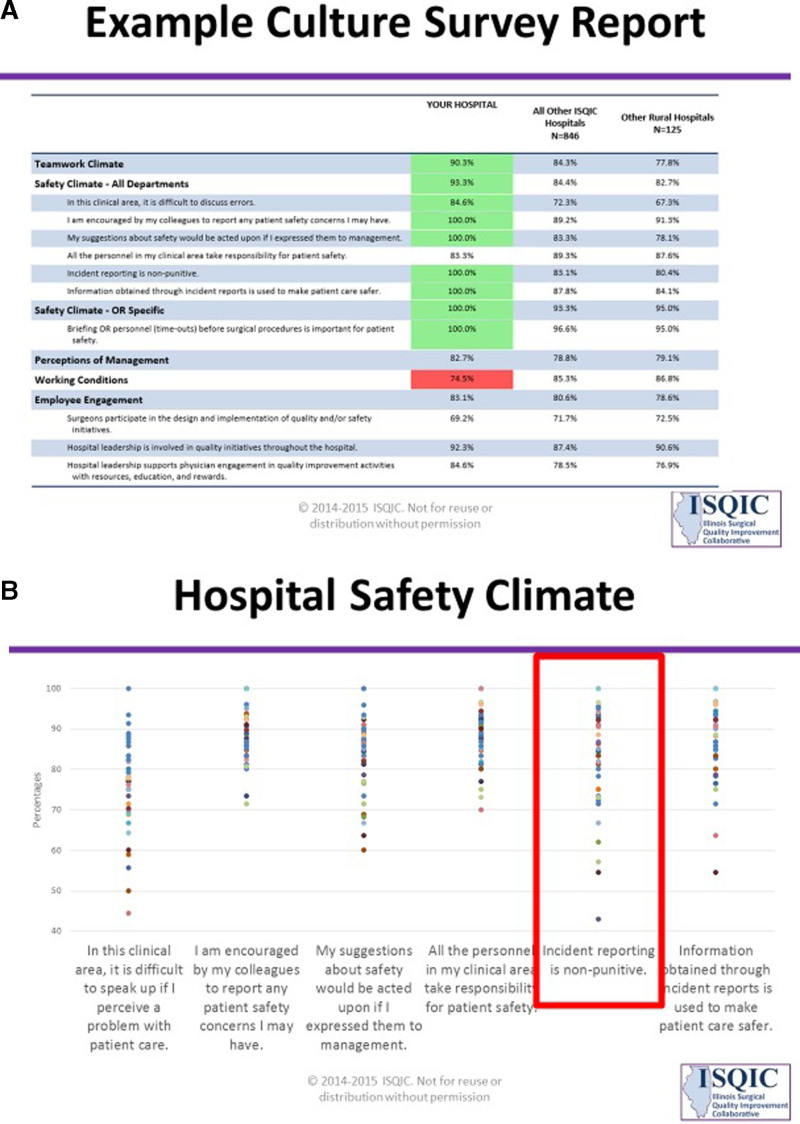
ISQIC QI and Safety Culture Comparative Report. A and B, ISQIC hospital safety culture report. OR, Operating Room.

To better understand empirically observed cultural variations toward QI and safety among ISQIC’s diverse hospital network, a surgery-focused Safety Attitudes Questionnaire (SAQ) was administered during years 1 and 3 to all participating hospitals. The SAQ helped the collaborative gauge the teamwork and safety climate at each hospital, as well as employee engagement and perception of management and the hospital’s SC. Hospitals received aggregate, blinded results comparing their hospital to the other ISQIC hospitals whereby allowing sites to objectively measure their QI culture and potentially lobby for additional QI/PI resources to improve engagement and culture (Fig. 5). Along with the ISQIC SAQ, hospitals were asked to designate either their Chief Quality Officer or Director of Quality to participate in an ISQIC QI Resources Assessment. This survey helped to assess the types of QI/PI resources available at each ISQIC hospital, in addition to assessing the hospital Board’s engagement in quality.

Final analysis composed of over 1000 respondents representing 36 ISQIC hospitals revealed important improvements in hospital safety culture between years 1 and 3.^41^ All SAQ domains increased within ISQIC hospitals, particularly within areas involving perceptions of teamwork and safety. Several hospitals experienced improvements in 1 domain, while, most hospitals experienced improvement across several SAQ domains. Noteworthy improvements were also observed within SAQ domains for hospitals, with respondents highlighting improved physician/nurse collaboration and surgeon participation in QI initiatives. Interestingly, hospitals with the lowest safety culture scores in year 1 experienced the greatest cultural improvements by year 3, with the difference attributed to ISQIC involvement. These findings remain particularly relevant since higher SAQ scores were associated with lower risk of postoperative morbidity, death or serious morbidity within ISQIC.^42^

#### Return on Investment Reports

**FIGURE 6.**
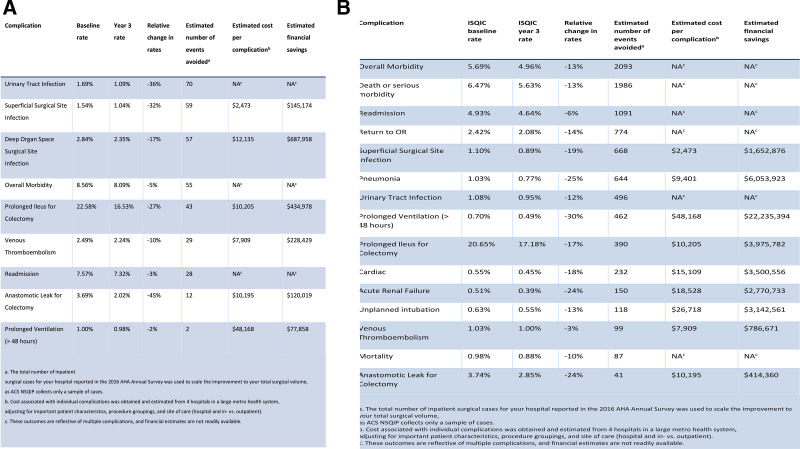
ISQIC hospital ROI reports. A, Reduction in surgical complications and estimated financial savings. B, ISQIC statewide improvement. AHA, American Hospital Association; NA, Not Applicable.

Following sufficient accumulation of 3 years of data, ISQIC hospitals were provided return-on-investment (ROI) reports (Fig. 6). These reports included: (1) each individual hospital’s ROI results, estimating the number of avoided complications and the associated financial savings based on each hospital’s NSQIP data, (2) collaborative-wide results showing similar complication avoidance and fiscal impact across ISQIC hospitals in aggregate, and (3) modeled hospital-level costs of NSQIP and ISQIC, demonstrating low participation costs compared to considerable savings. The ROI reports were intended to provide local hospital leaders objective evidence on the excellent ROI of ISQIC participation and QI.

### Domain 3: Guided Implementation of QI Methodology

#### Surgeon Mentor

Formally trained and experienced surgeon mentors were assigned to each hospital to guide and advise the hospital’s SC and ISQIC team through each phase of the program. Surgeon mentors were chosen from a pool of national ACS NSQIP SCs who successfully implemented QI projects at their hospital and applied to participate in the ISQIC mentorship program.^43^ Mentors underwent ISQIC training at the annual ACS NSQIP meeting and had access to all training and support tools that the hospitals received. Surgeon mentors were obligated to meet their assigned SC (or hospital quality team) via phone quarterly and meet with the hospital teams in person at the annual ACS NSQIP conference. In addition to the requisite interactions, some mentors voluntarily visited their mentee hospital teams in person. Surgeon mentors were available to answer questions about organizing a QI program, ACS NSQIP operations, leadership, change management, motivation, engagement, and how to influence change in surgical culture.

#### Process Improvement Coach

Local hospitals teams were assigned PI coaches as an external consultant to help teams navigate QI issues, implement improvements, and meet deliverable deadlines. PI coaches were experienced in healthcare systems quality and project management and typically were employed at regional hospitals in a non-ISQIC capacity. PI coaches guided and supported local teams through DMAIC methodology in conjunction with formal DMAIC didactic training.^39^ The ISQIC Coordinating Center provided salary support to a cadre of 10 PI coaches that were assigned 3–5 hospitals each over the collaborative’ s first 3 years. Through 6 bi-monthly requisite calls each year, PI coaches and local teams discussed QI projects, QI challenges, and potential remedies. Some of the PI calls were held with multiple hospitals present, whereby enabling hospital peers to share successes and challenges, exchange best practices and ideas, and build camaraderie.

Centrally, the ISQIC coordinating center held a monthly in-person meeting with all PI coaches. During the check-in, coaches briefed the coordinating center on how each hospital was fairing with QI projects, DMAIC methodology, local engagement, and identified if focused ad hoc interventions were necessary to remedy any areas of concern. The coordinating center was then able to provide coaches and teams focused interventions to assist teams with their challenges. A formal, shared tracking document was updated by coaches each month to centrally monitor progress for hospitals over time and detail operational milestones, barriers and remedies. The transparency of the tracking document provided an indirect incentive for hospitals to complete ISQIC projects and tasks.

#### Annual Statewide Collaborative Quality Improvement Project

Following nearly 1 year of site training and team building, every ISQIC hospital participated in a requisite annual CQIP aimed to improve surgical quality across the state (Table 2). Starting toward the end of the first year of the collaborative, 2 CQIPs (one for large hospitals, the other for small hospitals) were typically chosen annually by the ISQIC advisory committee based upon quality needs assessments of participating hospitals and discussions with the collaborative as a whole. Each year’s CQIPs required all similar-sized hospitals to simultaneously complete one shared specific initiative, typically over a 12–18-month span. CQIPs were introduced to the collaborative through introductory webinars over several months to allow local hospitals to orient to the problem, understand data definitions, and develop DMAIC-based implementation strategies.

**TABLE 2.** ISQIC Statewide QI Projects

Years	Large/Current Hospitals	Small-Rural Hospitals	Pediatric Hospitals
1	Composite VTE prophylaxis		
2	Implementation of a VTE intervention	Perioperative glycemic control	Appropriateness of blood transfusions
3	Surgical site infection reduction	Quality of colonoscopy	Uncomplicated appendectomy
4*	Improving surgical care and recovery (enhanced recovery after surgery)		
	Extended post-discharge VTE chemoprophylaxis		
	Decreasing Opioid prescribing after surgery		
	Video-based coaching		
5	Prehabilitation optimization		
6	Surgical cancer quality		
7†	Reducing catheter-associated urinary tract infection		
	Using Operating Room (OR) video for quality improvement		
	Establishing guidelines for retriage in trauma surgery patients		

*Switched to offering a menu of project options.

†Current/upcoming year.

CQIPs began with baseline process measure data collection typically for 2–3 months. Following analysis of baseline data, hospitals identified local failure modes and brainstormed solutions. After baseline data analysis, ISQIC developed a “toolkit” containing CQIP-specific implementation aids, tips, case studies, frequently asked questions, and learned best practices from early adopter hospitals. The goal was to provide hospitals with turnkey interventions to improve implementation fidelity. Since each hospital works in its own unique microenvironment, hospitals needed to tailor interventions to suit their local context. Surgeon mentors and PI coaches served as expert resources to ensure successful CQIP implementation and progress. The synchronous nature of many ISQIC hospitals working in parallel on a solitary project conveyed tremendous opportunity for shared learning among sites during periodically scheduled meetings, discussion board posts, and email list exchanges during the year-long-project. Resources created by 1 hospital would often be shared with the others, thus creating a very collaborative atmosphere.

#### Comprehensive Postoperative VTE Prophylaxis

The first CQIP focused on comprehensive postoperative VTE prophylaxis and was required by all small and large ISQIC hospitals.^44^ The CQIP was initiated mid-way through the collaborative’s first year and extended into the second year for large hospitals. The CQIP focused on a novel composite measure comprised of 3 guideline-recommended components of VTE prophylaxis^45^: (1) early ambulation, (2) mechanical prophylaxis (sequential compression devices), and (3) chemoprophylaxis. Correct chemoprophylaxis administration required defect-free care where the prophylaxis must have been given at the correct dose and frequency throughout the entire hospital stay with no missed doses. Following baseline data collection, hospitals analyzed local data to determine variations and target areas to improve as the ISQIC VTE Prophylaxis Toolkit was released. The toolkit, parts of which were co-developed with the Johns Hopkins hospital VTE team, provided hospitals known solutions to improve VTE prophylaxis with patient-, nurse-, and physician-specific implementation aids.^46^

**FIGURE 7.**
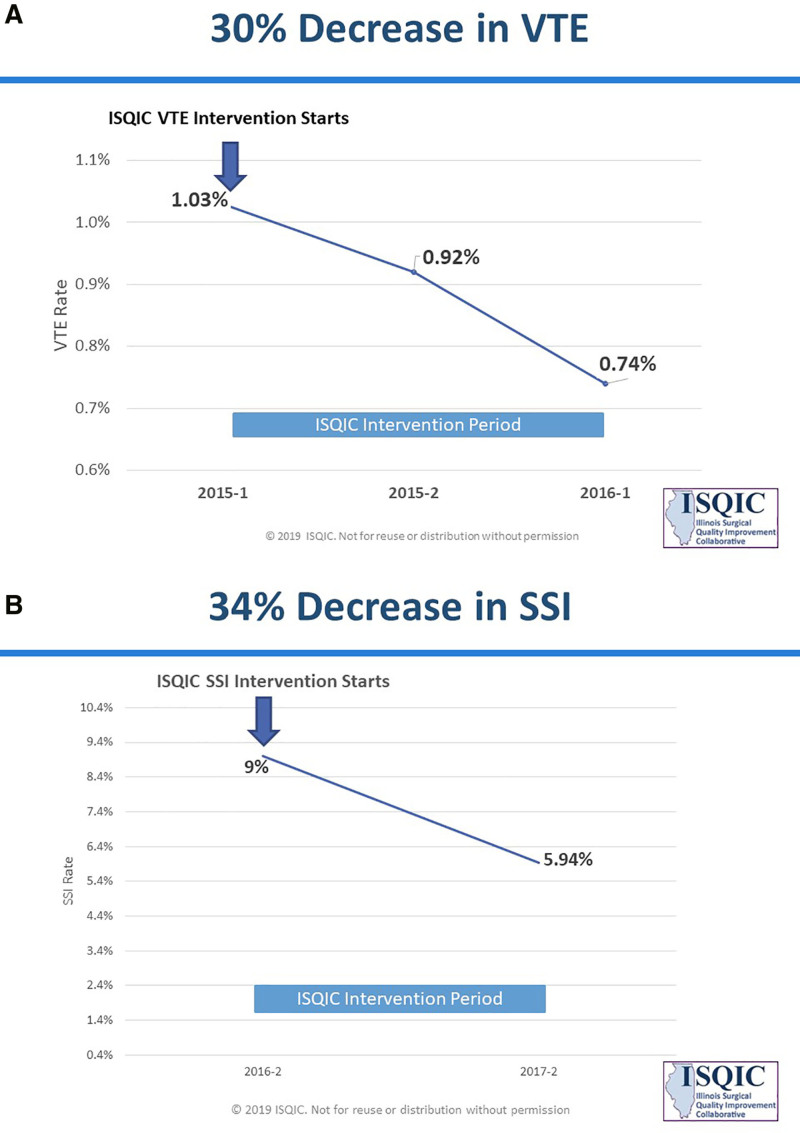
Improvement in ISQIC outcomes. A, VTE. B, SSI.

The CQIP provided powerful insights into VTE prophylaxis miscues across the state. CQIP data showed that VTE chemoprophylaxis was missed in up to 18% of patients in ISQIC, with failure to prescribe (30%), patient refusal (30%) and incorrect dose/frequency (8%) among the most common reasons for chemoprophylaxis failures.^44^ Qualitative analysis at 1 ISQIC hospital revealed that several nurse-based interventions could improve chemoprophylaxis administration.^47^ Importantly, VTE frequency correlated with numbers of missed chemoprophylaxis doses.^48^ As a result, the ISQIC VTE intervention reduced postoperative VTE rates across the state by 30% (Fig. 7A).

#### SSI Reduction Bundle

The second CQIP was limited to large ISQIC hospitals and focused on implementation of a perioperative SSI reduction bundle for colorectal resections. The bundle was developed based on contemporary evidence and best reported practices and followed the same CQIP cadence of pre- and post-intervention data collection. A robust ISQIC SSI toolkit was disseminated containing a curated mix of ISQIC-produced and borrowed resources from current literature and other Illinois hospitals containing pragmatic tips to further assist sites with implementation. The toolkit was intended to provide targeted implementation strategies for specific SSI reduction bundle barriers, which were then tailored to the local needs of each hospital.^49^

Among 32 participating large ISQIC hospitals, the colorectal SSI CQIP resulted in a 2.5-fold relative increase (20% vs 50%) in the number of patients completing at least 75% of the bundle. Adjusted analyses showed a trend toward lower risk of superficial incisional SSI in the post-implementation period compared to baseline with a 30% relative risk reduction in superficial SSI (Fig. 7B).^49^ Notably, as the adherence in the number of bundle elements increased, there was a linear significant decrease in SSI rates. Bundle adherence was increased for patients with higher BMI and reduced for ISQIC safety net hospitals.^50^ Variation in adherence rates across ISQIC was primarily attributable to hospital and patient factors, rather than surgeon factors. The large number of ISQIC patients allowed identification of specific bundle elements that were associated with SSI reduction.^51^ By allowing hospitals to intensify implementation of more effective SSI bundle elements and eliminating lesser effective measures, a leaner, more potent SSI bundle is planned for reissue to the collaborative.

#### Small-Rural Hospital CQIPs

Small and rural ISQIC hospitals typically perform outpatient or comparatively lower complexity surgeries when contrasted with larger hospitals. Following the collaborative-wide VTE CQIP for all ISQIC hospitals, subsequent, separate CQIPs were designed for small and rural hospitals that target their specific QI needs. This was an important lesson learned: projects for larger hospitals did not resonate with the small-rural hospitals. Following discussions with each small/rural hospital and determination of best and evidence-based practices, the first CQIP strictly for small and rural hospitals focused on preoperative screening for hyperglycemia for high-risk individuals. Small and rural hospitals would screen men over age 40 without history of diabetes mellitus with a single blood glucose at least 90 days before a surgery, and then reflexively check hemoglobin A1C if elevated. The measure also included checking a pre-operative hemoglobin A1C on patients known to have diabetes mellitus. An ISQIC CQIP Toolkit provided to hospitals educated providers to the importance of perioperative euglycemia and turnkey examples of how to implement systems to screen patients. Due to small patient volumes in small/rural ISQIC hospitals, this CQIP continues and is close to having sufficient data volume to power planned analyses.

The second dedicated small/rural hospital CQIP focused on the quality of colonoscopy. Unlike large and urban hospitals where gastroenterologists perform the majority of endoscopy, small and rural healthcare settings may lack enough gastroenterologists to meet endoscopy needs. As a result, endoscopy is the most commonly performed procedure for rural general surgeons.^52^ Aligned with gastroenterology societal recommendations, basic colonoscopy quality measures were developed.^53^ To improve the quality of rural colonoscopy, rates of successful cecal intubation and photo documentation of the cecum, appendiceal orifice, and rectal retroflexion during colonoscopy were audited and fed back to surgical endoscopists. As with other small-rural ISQIC projects, the relatively small volumes of endoscopies and small/rural hospitals have implicated data collection and the project continues as future data analysis is planned.

#### Pediatric Hospitals

Three pediatric hospitals joined ISQIC. Given their unique patient population, they did not participate in the same annual statewide CQIP projects. Instead, these 3 hospitals were treated as their own mini-collaborative and took on their own projects using the model of ISQIC. For example, 1 project sought to implement a same-day appendectomy discharge program across all 3 hospitals.

#### Annual Hospital-Specific Quality Improvement Project

In addition to CQIPs, ISQIC hospitals were required to complete 2 local QI projects each year. Local projects were based on a self-identified improvement need based on their Illinois-specific benchmarked quality reports (Fig. 4). While hospitals were encouraged to develop individualized projects to suit their needs, ISQIC provided 2 projects for hospitals who did not identify a compelling local need. The first optional local project focused on ensuring appropriateness of perioperative blood transfusions. Participating hospitals monitored occurrences of perioperative blood transfusions and were provided with target transfusion thresholds. The second optional local project focused on post-discharge VTE chemoprophylaxis for orthopedic and abdominal oncologic surgeries. To support hospitals focusing their local projects on these topics, toolkits were developed and coaching calls conducted.^41,43,49^

### Domain 4: Networking

Semiannual in-person ISQIC conferences were held to conduct ISQIC business and facilitate networking and sharing of projects, resources, and ideas (Supplemental Figure 3A, B, http://links.lww.com/AOSO/A209). Local hospital teams were required to attend the meetings. The meetings were intended to provide updates on local QI projects, as well as to participate in training, education, idea sharing, and team sharing exercises. The meetings provided an opportunity to discuss and launch statewide collaborative projects for the upcoming year and hear progress feedback on current initiatives. A standing meeting venue was selected in the far west suburbs of Chicago for these conferences to ease travel for geographically diverse teams. Meeting formats differed from meeting to meeting in order to keep the content and format fresh for participants. While there was a keynote speaker each meeting, the remainder of the meeting included multiple components including lectures, small group discussions, workshops, impromptu discussion topics, and a variety of other formats.

In advance of conferences, local teams were asked to complete preparatory exercises to prime attendees and enhance engagement and efficiency during the meeting. Several strategies were employed by meeting organizers to increase participant engagement and interaction including: role-playing scenarios, best-practice discussions, crowd-sourced topic generation, small focus group discussions, and academic poster sessions detailing local QI projects. Networking and cross-pollination was prioritized through orchestrated working lunches where groups working on similar projects sat together to synergize efforts. Conferences also included long casual coffee breaks and less structured time to allow organic social networking to create a sense of community and camaraderie.

#### Webinars

During ISQIC’s first 3 years, over >30 webinars allowed hospital teams to discuss barriers and generate solutions, idea-share, and collaborate pertaining to a diverse range of topics. While most webinars focused on CQIP projects, supplementary and ad hoc meetings were held to promote continued collaboration during lulls between meetings. Webinars were hosted by the ISQIC Coordinating Center, and depending upon meeting content speakers would include individuals from participating ISQIC hospital teams, national experts, and coordinating center leaders and staff.

#### ISQIC Coordinating Center Site Visits

An ISQIC-led interdisciplinary team of researchers, physicians, PI experts, and coordinating center staff conducted site visits to assess hospitals’ QI capabilities, to explore factors influencing the adaptation of ISQIC components and to improve the collaborative methodology for a sample of participating hospitals. A site visit protocol, questionnaire, and scoring rubric based on the 21 components was developed and iteratively refined to support data collection and analysis from observations and interviews. Twenty-five hospitals were visited and meetings included focus group discussions and semi-structured interviews with the hospital’s SC, SCR, Chief Medical Officer, Director of Quality, frontline surgeons and nurses, and trainees. At each hospital, the site visit team identified: (1) strengths and exemplary practices, (2) opportunities, concerns, or vulnerabilities, and (3) valuable findings to share collaborative-wide. Site visits conducted according to an iteratively refined protocol allowed ISQIC hospitals to receive feedback regarding their surgical QI program, culture, and adaptation of ISQIC components. In turn, the visits allowed ISQIC to identify local challenges first-hand and to promote the work of the local ISQIC team to local hospital leaders.

### Domain 5: Financial Support

#### Financial Support to Individual Hospitals

BCBS-IL contracted with each funding-eligible hospital to provide a stipend to support the costs of collaborative participation for the first 3 years. The stipend covered the cost of the ACS NSQIP participation fee (~$25,000), the collaboration participation fee, support for the SCR salary, a SC and mentor stipends, and travel to conference meetings. Importantly, the BCBS-IL stipend was closely tied to deliverables which were documented and verified bi-annually in order for hospitals to get the subsequent year’s funding. In year 3 of ISQIC, hospitals were required to demonstrate a statistically significant improvement in at least 1 key surgical outcome around which they had an ongoing project in order to keep their financial support. Of note, all hospitals achieved this goal.

#### Pilot Grants

ISQIC included a pilot grant proposal and funding mechanism to fund selected hospitals’ costs for local QI initiatives. Hospitals that identified an issue requiring funding submitted a competitive application for additional grant support. Grants ranged from $5000 to $25,000 and allowed hospitals to implement QI solutions that may have upfront costs involved that exceed current hospital budgets. For example, Delnor hospital proposed a project to decrease post-surgical catheter-associated urinary tract infections by using observations to identify and correct common failures. After being awarded an ISQIC pilot grant, they were able to purchase preoperative urine analysis kits and other catheterization materials leading to a significant decrease in their postoperative urinary tract infection rates. Hospitals were required to report back on their project at the ISQIC semiannual meetings and to submit an abstract on their project to the national ACS NSQIP conference.

## RESEARCH PLATFORM

In addition to innovative QI efforts, ISQIC was also leveraged as a platform for innovative research, using the 56 hospitals as a “learning health system” laboratory. More than 12 federal and private grants have utilized the ISQIC platform for research, simultaneously using the collaborative as a “laboratory” while bringing resources and innovation to ISQIC hospitals through the grant funding.

### Evaluation of Approaches to Quality Improvement

In 2016, the ISQIC coordinating center (PI: K.Y.B.) was awarded an Agency for Healthcare Research and Quality Health Services Research Projects (R01) grant to perform a detailed evaluation of the implementation of ISQIC. The grant focused on a rigorous mixed-methods evaluation of the implementation, adaptation, and effectiveness of ISQIC as well as examining ISQIC’s mechanisms for success and future sustainability. The ISQIC coordinating center conducted an extensive evaluation of the 21 components to understand which parts and how well each of the individual components contribute to the goal of improved surgical safety and cost savings. Specifically, the evaluation effort has focused on (1) identifying site-specific differences in the implementation and adaption of ISQIC, (2) barriers and facilitators to implementation & improvement, (3) intensity of implementation of ISQIC, and (4) effects of organizational climate, attitudes, engagement and culture on quality of care. Data collection included site visit documentation, artifact analysis, and other questionnaires and surveys throughout the first 3 years of the collaborative (Supplemental Table 2, http://links.lww.com/AOSO/A209).

### Examples of Other Grants

Numerous other grants were awarded to faculty in the ISQIC coordinating center that leveraged the collaborative as a laboratory and brought resources to support statewide projects. For example, federal and industry grants were awarded to support system-level efforts to reduce excess opioid prescribing in surgery and to preventing opioid misuse through agreements between patients and surgical providers (PIs: J.J.S., J.K.J.). Another example of a funding partnership with industry, grant funding was received to support ISQIC’s development of a prehabilitation optimization program to improve perioperative outcomes for elective colorectal patients in Illinois. Building upon principles used in the American College of Surgeons Strong for Surgery program, dynamic toolkits, collaborative conferencing, and guided implementation strategies were created by ISQIC and the ACS to support implementation of interventions in nutrition, smoking cessation, physical function, and cognitive preparedness at each hospital (PI: M.F.M.).

### Prospective Cluster-Randomized Trials

ISQIC CQIPs have allowed for prospective cluster randomized trials of QI and/or policy interventions. For example, in tandem with implementation of the colorectal SSI reduction bundle as the large hospital year 3 CQIP, we conducted a 2-arm, cluster-randomized pragmatic trial comparing the incremental effectiveness of providing physician-level feedback through electronic surgeon-specific and hospital-specific dashboards. The primary aim was to investigate whether implementing a surgeon-level SSI prevention electronic dashboard on top of ongoing local QI activities and data collection on the ISQIC SSI bundle increases the number of SSI prevention standards of care that colorectal surgery patients receive, compared to patients undergoing surgery in hospitals that do not have surgeon-level SSI prevention dashboards. While the intervention did not have an impact, there were several lessons learned about how to effectively provide individual-surgeon performance back to surgeons. The project also served as a role model for using ISQIC for pragmatic trials.

## FUTURE DIRECTIONS

After the third year of ISQIC in 2017, BCBS-IL start-up funding to participant hospitals expired. Remarkably, only 3 hospitals withdrew from ISQIC at the end of funding period, which confirmed the “starter program” philosophy had been effective in reducing upfront costs and allowing hospitals the opportunity to realize ISQIC’s value. Without funding, participation with ISQIC initiatives has become voluntary as hospitals do not have to meet BCBS-IL participation requirements. These changes will require ISQIC to adapt and evolve in several ways for continued success. Since hospitals now assume costs of ACS NSQIP membership, ISQIC will need to remain committed to the value proposition of membership; although a convincing evidence-based and financial argument can be made that ongoing ISQIC participation is prudent. Thus far, the argument seems to be correct given that nearly all hospitals have continued to participate in ISQIC.

## CONCLUSIONS

A novel, cost-effective, learning health system collaborative can be created with a diverse group of hospitals to successfully improve surgical quality in the state of Illinois. Not only was ISQIC responsible for demonstrable improvements in surgical quality for participant hospitals, but the collaborative yielded tremendous understanding of how hospitals can learn and collaborate effectively. The foundation provided by the first 3 years of ISQIC have cemented the collaborative as major contributor to surgical QI globally, and participant hospitals are poised to continue to deliver excellent quality surgical care.

## ACKNOWLEDGMENTS

K.Y.B., M.F.M., R.L., L.K., A.R.D., B.D., and A.D.Y. participated in research design, writing of the article, and performance of the research. M.V.W., J.K.J., A.L.H., K.J.O., P.F., J.T., S.R., K.D., M.S., Y.S., C.Q., V.P., K.A.C., D.O., J.J.S., C.B., J.H., and R.P.M. participated in research design and performance of the research. S.S., K.B., W.H., and C.F. participated in performance of the research.

## Supplementary Material


