# Supplementary material for: Development of the Illinois Surgical Quality Improvement Collaborative (ISQIC): Implementing 21 Components to Catalyze Statewide Improvement in Surgical Care
Source: Ann Surg Open. 2023 Mar 1;4(1):e258. doi: 10.1097/AS9.0000000000000258 (PMC9987591; doi:10.1097/AS9.0000000000000258)
Supplement: Supplementary file 1 [file as9-4-e258-s001.pdf]

Supplemental Digital Content

SDC, Table 1: ISQIC's 21 Strategies to Facilitate Quality Improvement

**ISQIC Components**	**Description**
Guided Implementation	
Surgeon Champion (SC)	Leads NSQIP and ISQIC initiatives for the hospital
Surgical Clinical Reviewer (SCR)	Nurse who performs data abstraction and manages QI projects
Surgeon Mentor	Surgeon Champion who has successfully lead ACS NSQIP elsewhere and serves as mentor for SC
Process Improvement (PI) Coach	Highly trained in PI to coach hospital QI teams through QI/PI projects
Coordinating Center (CC)	Provide leadership and support staff for all aspects of ISQIC implementation
Annual Statewide Collaborative Quality Improvement Project (CQIP)	QI project identified by ISQIC Advisory Committee to address statewide need. Carried out with assistance from Mentor, Coach and Coordinating Center.
Annual hospital-specific QI project	QI project identified by individual hospital QI team to address a specific area of poor performance.
Education	
Formal QI/PI curriculum	Formal process improvement training through online modules and in-person training sessions
Project Management Training	Training SCRs on effective project management skills
Hospital Board Engagement Program	Training and guidance for engaging the hospital's board in ISQIC initiatives and surgical QI
Best Practice Guidelines	Evidence-based best practices identified by expert panel
Surgical QI Case studies	Examples of how other NSQIP previously examined and addressed high rates of common postoperative complications

Toolkit for SC/SCR and Administrators	Step-by-step guide on how to be an effective SC/SCR and Administrator focused on QI
Comparative Reports	
Hospital-level risk-adjusted comparative data	Reports that allow *hospitals* to compare data on process of care and postoperative outcomes benchmarked against hospitals in Illinois and the U.S. Hospital-level return on investment reports are provided as well
Surgeon-level risk-adjusted comparative data	Reports that allow *surgeons* to compare data on process of care and postoperative outcomes benchmarked against hospitals in Illinois and the U.S.
Networking	
Conference Meetings	Three in-person conferences (2 ISQIC, 1 NSQIP) to facilitate sharing of experiences, work on common projects, and conduct process improvement training
Monthly webinars for SC/SCRs	Webinars to collaborate, share ideas, and trouble shoot issues
SCR-SC Meetings	Meetings scheduled to foster communication among hospital team, discuss cases, and implement QI/PI projects
Financial Support	
Stipend to hospital	Support for data abstractor, Surgeon Champion, NSQIP annual fee, travel to conferences, information technology, coordinating center, mentor, coach, comparative reports, pilot grants, PI curriculum, all resources
Pilot Grants for QI Projects	Hospitals may receive additional funding to implement related QI/PI projects
Bonus for improved outcomes	Financial bonus to hospitals that significantly improve outcomes by Year 3

SDC, Table 2: ISQIC Evaluation Approaches

Data Collection Tools	Focus of research	Analytic Approach
Site Visits: Ethnographic Observations, Semi-structured Interviews, Focus groups, Process mapping	Differences in site-specific implementation of ISQIC, barriers and facilitators to implementation, barriers and facilitators to improvement, culture for high quality care	Qualitative analysis of key themes, development of hospital specific case studies, cross case comparison
Artifact Analysis: ISQIC Application, Existing resources survey, Letters of support, Case volume worksheet, Progress reports, Documentation of mentor calls, Documentation of PI coach calls, Pilot grant application, Board powerpoint presentation, SC-SCR meeting form, CQIP Project Plan Form, Other site specific materials for implementation collected during site visit	Intensity of implementation of ISQIC, uptake and adaptation of ISQIC components	Key themes, case study, cross case comparison
Surveys: Safety Attitudes Questionnaire (SAQ), Quality Improvement Knowledge Assessment Test (QIKAT), Board Engagement Survey, Mentor evaluation, PI Coach Evaluation	Organizational climate, Knowledge, attitudes and skills necessary for quality improvement activities, board engagement, mentor effectiveness, evaluation of hospital engagement	Descriptive analysis, pre-post tests

SDC, Figure 1. ISQIC Hospital QI Team

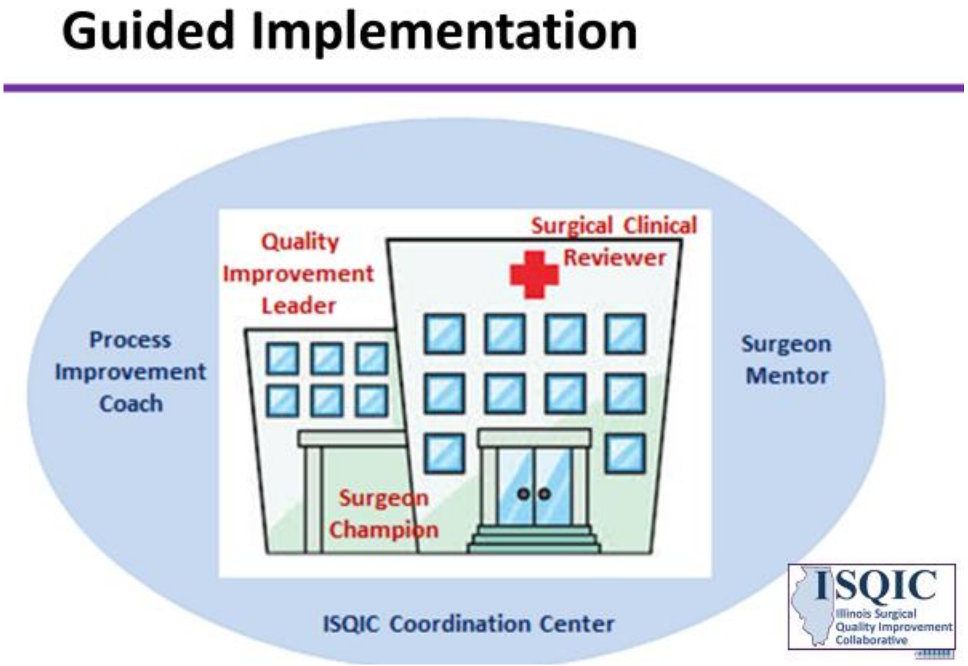

SDC, Figure 2. ISQIC Data Platform

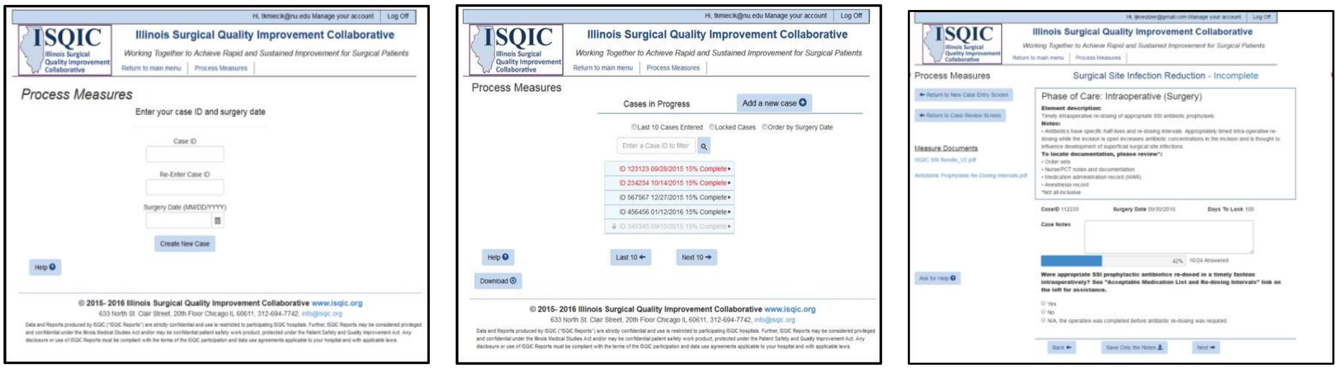

SDC, Figure 3a-b: ISQIC Semiannual Conference Agendas

ISQIC Annual Meeting Agenda<br>Friday, January 22, 2016			
**Time**	**Section Title**	**Lead Faculty**	**Location**
8:00am-8:30am	Check-In and Breakfast		Lobby
8:30am-9:00am	Opening Remarks and Updates	Karl Bilimoria	Auditorium
9:00am-10:30am	Interactive Hospital Project Presentations	Mark Schumacher, Tony Yang & Hospitals	Auditorium
10:30am-10:45am	Break		
10:45am-12:00pm	Keynote and Discussion: Dr. Michael Englesbe, Associate Director for the Michigan Surgical Quality Collaborative (MSQC)		Auditorium
12:00pm-1:20pm	Networking Lunch		Ballroom
1:20pm-1:30pm	Travel		
1:30pm-2:30pm	Crowd-Sourced Discussions		Breakout Rooms
Topic: VTE<br>Leader: Todd Petty (Blessing Hospital)		Facilitator: Joseph Caprini	Room 260
Topic: SSI Reduction<br>Leader: David Olysav (Abraham Lincoln Memorial Hospital)		Facilitator: David Odell	Room 261

<table>
<tr><td colspan="3">Topic: ERAS

Leader: James Girardy (OSF) **Facilitator: Jonah Stulberg**</td><td>Room 265</td></tr>
<tr><td colspan="3">Topic: Sustainability of Improvements

Leader: Sue Sullivan (The University of Chicago) **Facilitator: Steve Reinhart**</td><td>Room 266</td></tr>
<tr><td colspan="3">Topic: Building Engagement/Buy-In

Leader: Carol Schultz (Presence HealthSystem) **Facilitator: Amy Halverson**</td><td>Room 250</td></tr>
<tr><td colspan="3">Topic: Barriers to QI

Leader: Linda Cooper (OSF St. Francis) **Facilitator: Julie Johnson**</td><td>Room 256</td></tr>
<tr><td>**2:30pm-2:50pm**</td><td colspan="3">Travel & Break</td></tr>
<tr><td>**2:50pm-3:20pm**</td><td>Crowd-Sourcing Debrief</td><td>Kevin O'Leary</td><td>Auditorium</td></tr>
<tr><td>**3:20pm-3:35pm**</td><td>New Video-Based Coaching Project</td><td>Karl Bilimoria/Jonah Stulberg</td><td>Auditorium</td></tr>
<tr><td>**3:35pm-4:00pm**</td><td>Exciting Changes to ISQIC</td><td>Karl Bilimoria</td><td>Auditorium</td></tr>
<tr><td>**4:00pm-6:00pm**</td><td colspan="2">**Cocktail Reception**

&

Presentation of ISQIC Excellence in Quality Awards</td><td>Ballroom</td></tr>
</table>

ISQIC Annual Meeting Agenda
Friday, January 20, 2017

Time	Section Title	Moderator	Location
**8:00am-8:30am**	Check-In and Breakfast		Lobby
**8:30am-9:00am**	Opening Remarks & ISQIC Updates	Karl Bilimoria	Auditorium
**9:00am-9:30am**	Curtailing the Opioid Epidemic in Illinois	DEA Special Agent in Charge: Dennis Wichern	Auditorium
**9:30am:10:15am**	Surgeons and the Opioid Epidemic: ISQIC Panel Discussion<br>1. Swedish American Hospital<br>2. University of Chicago Medical Center<br>3. Northwestern Memorial Hospital	Vivek Prachand & Jonah Stulberg	Auditorium
**10:15am-11:15am**	Break and Hospital Quality Poster Discussion		Ballroom
**11:15am-12:15pm**	Keynote Presentation: Clifford Ko, MD, MS, MSHS, FACS, FASCRS		Auditorium
**12:15pm-1:30pm**	Lunch		Ballroom
**1:30pm-2:30pm**	ISQIC Pilot Grant Recipient Presentations<br>1. Memorial Hospital of Carbondale-ERAS<br>2. Herrin Hospital-Glycemic Control<br>3. Delnor Hospital – UTI Project	John Wieland & Tony Yang	Auditorium
**2:30pm-2:45pm**	CQIP: SSI Reduction Bundle Developments	Michael McGee	Auditorium

2:45pm-3:15pm	Video-Based Coaching Initiative & Live Video Scoring	Jonah Stulberg	Auditorium
3:15pm-3:30pm	Announcements and Awards	Karl Bilimoria	Auditorium
